# Prevalence of Lower Extremity Arterial Disease as Measured by Low Ankle-Brachial Index in Patients with Acute Cerebral Ischemic Events

**DOI:** 10.3390/jcm9103265

**Published:** 2020-10-12

**Authors:** Magdalena Konieczna-Brazis, Paweł Sokal, Paweł Brazis, Tomasz Grzela, Milena Świtońska, Violetta Palacz-Duda

**Affiliations:** 1 Department of Neurosurgery and Neurology, Jan Biziel University Hospital No. 2, Ujejskiego 75 Street, 85-168 Bydgoszcz, Poland; magdabrazis@gmail.com (M.K.-B.); m.switonska@cm.umk.pl (M.Ś.); violkapduda1@tlen.pl (V.P.-D.); 2 Faculty of Health Sciences, Collegium Medicum in Bydgoszcz, Nicolaus Copernicus University in Toruń, Jagiellońska 13-15, 85-067 Bydgoszcz, Poland; 3 Department of Vascular Surgery and Angiology, Dr Jurasz University Hospital No. 1, Collegium Medicum Nicolaus Copernicus University, Marii Skłodowskiej Curie 9 Street, 85-094 Bydgoszcz, Poland; mpbrazis@wp.pl; 4 Department of Vascular Surgery, The 10th Military Research Hospital, Powstanców Warszawy 5 Street, 85-681 Bydgoszcz, Poland; tomg@byd.net.pl

**Keywords:** stroke, TIA, lower extremity arterial disease, ankle–brachial index

## Abstract

Background: Low ankle–brachial index (ABI) of ≤0.9 is diagnostic of lower extremity arterial disease (LEAD). It is also a strong marker of generalized atherosclerosis. The objective of this study was to assess the prevalence of low ABI in patients with acute cerebral ischemic events (ACIE): ischemic stroke (IS) or transient ischemic attack (TIA). Methods: We compared 150 inpatients with ACIE to 50 inpatient controls and assessed risk factors, ABI measurements, and Duplex ultrasound of the cervical vessels. Results: Low ABI was seen in 69 patients (46%) in the ACIE group and in 8 (16%) in the control group; *p* < 0.01. The mean and median ABI values in the ACIE group were 0.88 (SD = 0.22) and 0.91 (0.24–1.33), which were significantly lower than in the control group: 1.04 (SD = 0.16) and 1.0 (0.66–1.36); *p* < 0.0001, respectively. Coronary artery disease, carotid stenosis of ≥50% and smoking were risk factors, which were associated with significantly lower ABI in the study group; the ABI with risk factors vs. without was 0.85 vs. 0.92 (coronary artery disease); *p* < 0.05, 0.7 vs. 0.92; (carotid stenosis) *p* < 0.001 and 0.83 vs. 0.98; (smoking) *p* < 0.001, respectively. Conclusion: Our study demonstrated that patients with ACIE have significantly higher involvement of another vascular bed as LEAD. Coronary artery disease, carotid stenosis ≥50% and smoking were main risk factors associated with coexistence of LEAD and ACIE.

## 1. Introduction

Atherothrombosis may occur in any arterial territory. Frequently, it is present simultaneously in more than one arterial bed. Multisite artery disease (MSAD) is defined as a presence of clinically relevant atherosclerotic lesions in at least two major vascular beds. According to the 2017 Guidelines on the Diagnosis and Treatment of Peripheral Arterial Diseases, in collaboration with the European Society for Vascular Surgeons, the term “peripheral arterial disease” (PAD) encompasses all arterial diseases other outside of the coronary arteries and the aorta. Previously, the term PAD had been used only for atherosclerotic processes of the lower limbs. The ABI (ankle–brachial index) is a recommended tool for clinical practice and research according to the European Society of Cardiology guidelines. ABI is a non-invasive, first-line test for screening and diagnosis of LEAD. An ABI ≤0.9 is diagnostic of LEAD [1]. A low ABI is a strong marker of generalized atherosclerosis and cardiovascular risk [2,3,4]. LEAD is also a known independent predictor for stroke [5]. Low ABI increases the risk for TIA and stroke two- to fourfold [4,6]. The majority of patients with LEAD are asymptomatic [6,7,8,9]. Nevertheless, they still have increased risk of cardio- or cerebrovascular events and death as do patients with symptomatic LEAD [10,11]. ABI is a simple and accurate test which should be included in routine screening of cardiovascular status [10] and is reliable, objective tool to detect LEAD and predict stroke [12,13]. The objective of this study is to assess the prevalence of a low ankle–brachial index (ABI ≤ 0.9) in patients with acute cerebral ischemic events (ACIE): ischemic stroke (IS) or transient ischemic attack (TIA).

## 2. Experimental Section

One hundred and fifty inpatients with IS or TIA (stroke group) and 50 within the control group were evaluated in the Stroke Interventional Treatment Center of the Department of Neurosurgery and Neurology. The control group consisted of neurological inpatients with epilepsy, multiple sclerosis, or vertigo, without diagnosis of acute cerebral vascular disease.

The study was conducted according to the rules of the Helsinki Declaration and requirements of the local ethics committee. Permission No 486/2011 was granted by Bioethics Committee of the Nicolaus Copernicus University in Bydgoszcz. Each patient was informed about the study and signed consent to participate. The trial has been registered in the appropriate registry—ClinicalTrials.gov Identifier: NCT03948399. The ACIE group included patients with IS or TIA of the anterior cerebral circulation. Exclusion criteria were: primary intracranial hemorrhage, venous sinus thrombosis, intubation, and inability to provide informed consent. Patients underwent either computed tomography (CT) or magnetic resonance imaging (MRI) of the brain, duplex ultrasound (DUS) of cervical vessels, and assessment of stroke risk factors. Detailed criteria for diagnosis of internal carotid artery (ICA) stenosis thresholds using peak systolic velocity (PSV), end-diastolic velocity (EDV), and their ratios in the ICA and common carotid artery (CCA) were based on the North American Symptomatic Carotid Endarterectomy Trial (NASCET) method [14]. Hypertension was defined as a systolic BP ≥140 mmHg and a diastolic BP ≥90 mmHg, measured at least twice. Atrial fibrillation was diagnosed by either electrocardiography (ECG) or Holter ECG. Coronary artery disease was diagnosed by ECG, echocardiography, medical history, and confirmed by consulting cardiologist. Diabetes mellitus was recognized as fasting blood glucose ≥126 mg/dL, a positive oral glucose tolerance test, current insulin therapy, or oral hypoglycemic agents. Hyperlipidemia was defined as a serum total cholesterol ≥200 mg/dL, low-density lipoprotein (LDL) cholesterol ≥135 mg/dL, high-density lipoprotein (HDL) cholesterol <40 mm/dL, and triglycerides ≥150 mg/dL. Participants were considered current smokers if they smoked cigarettes before the admission or as previous smokers if their smoking cessation was at least 5 years ago. Each participant underwent an ABI measurement. ABI was measured in the supine position, with the cuff placed above the ankle. After a 10 min rest, the SBP (systolic blood pressure) was measured by a Doppler probe (8 MHz) on the posterior tibial and dorsalis pedis arteries of each foot and on the brachial artery of each arm. The ABI of each leg was calculated by dividing the highest ankle SBP by the highest arm SBP. For statistical interpretation we took the lowest ABI between the legs of each subject. The diagnosis of LEAD was made if the ABI value was ≤0.9 according to the ESC Guidelines. Interpretation of ABI was as follows: ≤0.9 = low; 0.91–1.39 = normal; and ABI > 1.4 = high.

### Statistical Analysis

Data were analyzed in STATISTICA SOFTWARE 13.0 using non-parametric tests, such as Mann–Whitney U test, Kruskal–Wallis test, Chi-square test with the appropriate correction depending on the number of groups. A *p* value <0.05 was considered statistically significant for all comparisons.

## 3. Results

In our sample there were 119 (79.3%) patients with ischemic stroke (IS) and 31 (21.7%) patients with transient ischemic attack (TIA).

Demographics: The average age in the stroke group was significantly higher and amounted to 67.3 years in comparison to 63.36 years in the control group; *p* < 0.05.

Men constituted 60% in the study group and 40% in the control group; *p* < 0.05. Table 1.

### 3.1. Risk Factors

Table 2 presents risk factors for acute cerebral ischemic events in both groups. Previous ACIE was found more frequently in the stroke group than in the control group; 33 (22%) vs. 4 (8%); *p* < 0.05. Among co-morbidities, coronary artery disease was significantly more common in the stroke group than in the control group, 44 (29.3%) vs. 4 (8%); *p* < 0.01. The stroke group also had a higher number of hypertensive patients—132 (88%) vs. 28 (56%) in the control group; *p* < 0.0001. Among these patients, the difference between the subgroup with untreated hypertension in the stroke group compared to the control group was significant; 42 (28%) vs. 3 (6%); *p* < 0.05. The differences in the subgroup with treated hypertension were not statistically significant. In the stroke group there were more patients with atrial fibrillation: 29 (19.33%) vs. 1 (2%); *p* < 0.01 and diabetes 56 (37.33%) vs. 6 (12%); *p* < 0.001. Patients with carotid stenosis were divided into significant ICA stenosis ≥ 50% and hemodynamically non-significant <50% stenosis. 113 patients (75.33%) and 37 (24.67%) were found in the stroke group, and 50 patients (100%) and 0 (0%) in the controls; *p* < 0.00, respectively. A significant difference in HDL level was observed in the fasting lipid profile; HDL was lower in the stroke group than in the control group; *p* < 0.001. There was no significant difference between the groups concerning the overall cholesterol levels, triglycerides or LDL levels. Additionally, statins usage was assessed. In both groups their percentage was the same, stroke group 63 (42%) vs. controls 21 (42%), not significant (NS). Current smoking status was more prevalent in the stroke group than in the controls, 48 (32%) vs. 8 (16%); *p* < 0.05. Table 2.

### 3.2. Distribution of Normal and Low Values of ABI in the Stroke and Control Groups

Low ABI (≤0.9) was found in 69 subjects (46%) in the stroke group and in 8 (16%) in the control group, *p* < 0.01. Normal ABI (0.91–1.39) was seen in 69 patients (46%) in the stroke group, while in 37 patients in the control group (74%), *p* < 0.01. Table 3. The percentage of patients with high ABI >1.4 was similar in both groups: 12 patients (8%) in the study group vs. 5 patients (10%) in the control group; *p* = 0.44. This subgroup was excluded from further calculations.

### 3.3. ABI Values in the Stroke and Control Groups

The mean and median ABI values in the stroke group were significantly lower than in the control group; 0.88 (SD = 0.22) and 0.91 (0.24–1.33) vs. 1.04 (SD = 0.16) and 1.0 (0.66–1.36); *p* < 0.0001, respectively, Table 4.

### 3.4. ABI Values in the Stroke Group within the Subgroups with IS and TIA

When assessing the median ABI in the stroke group between patients with TIA and IS, no significant differences were found; 0.88 vs. 0.98; *p* = 0.2.

Median values of ABI depending on sex in both groups, differences among men and women were statistically significant: 0.88 vs. 1.08; *p* < 0.001 and 0.92 vs. 1.0; *p* < 0.01, respectively. Analyzing the groups with consideration to age, younger patients (≤65 years old) in the stroke group had ABI values significantly lower than in the control group, 0.9 vs. 1.0; *p* < 0.001. Older patients (>65 years old) in the stroke group had lower ABI values than in the control group, 0.92 vs. 1.07; *p* < 0.05. Table 5.

### 3.5. ABI Values Depending on Risk Factors and Stroke Subtypes in the Stroke Group

The presence of risk factors as a coronary artery disease, carotid stenosis ≥50% and smoking in the stroke group significantly decreases ABI value; 0.85 vs. 0.92; *p* < 0.05, 0.7 vs. 0.92; *p* < 0.001 and 0.83 vs. 0.98; *p* < 0.001, respectively (Table 6).

Among stroke subtypes the lowest ABI value was found in large-artery atherosclerosis stroke; 0.74. In subtype of undetermined etiology ABI value was also low; 0.9, (Table 7). 

## 4. Discussion

The goal of this study was to assess the prevalence of low ABI in ACIE. ABI ≤ 0.9 is diagnostic of LEAD, and it is also a good tool for risk assessment of cerebrovascular disease. It is important to identify populations at risk of having a low ABI in determining their future risk of ACIE. Investigation of risk factors for both LEAD and ACIE (IS and TIA) would allow to screen patients for ABI measurement. These patients might benefit from an early stroke prevention. In our sample, the median age was significantly higher in the stroke group than in the controls. This is explained by the fact that age is a recognized risk factor for IS, TIA, and LEAD [15]. In the Systemic Risk Score Evaluation in Ischemic Stroke Patients (SCALA) study, the mean age of stroke patients was 67 ± 12.4 years [16], which is similar to our study. In a review of population studies, the average age of stroke was 65 years or more in the range from 46.1 to 73.3 years [17]. These data are confirmed by the latest epidemiological stroke data, which states that two-thirds of patients hospitalized due to IS are over 65 years of age [18].

The incidence of IS is higher in men than in women [18], as seen in SCALA study where 57% of all patients were male [16]. Our sample was similar in sex characteristics. IS or TIA was more common in men (60%) than women (40%). Sex relationship in IS is unchanged in recent epidemiological data. Women, aged 45–84, have a lower stroke risk than men [18]. Our results of risk factors for ACIE proved to be the same as in the published literature [19].

### 4.1. Low ABI

It is known that the prevalence of low ABI in the general population ranges widely, from 3.7% to 28.7% [2,10,15,20]. In the current study, 46% of patients with ACIE had a low ABI, indicating frequent second vascular territory involvement. The percentage of low ABI was only 16% in the control group. According to available data, 9 to 51% of patients with acute ischemic strokes have concurrent LEAD [9,21,22,23,24,25,26,27]. Busch et al. observed 204 patients with IS or TIA over two years. Low ABI occurred in 61 patients (31%) [22]. In the PATHOS study, the authors examined 1758 patients admitted to the hospital due to acute coronary artery disease, IS or TIA. In the neurological group consisting of 755 patients, low ABI was seen in 34% of cases [23]. SCALA study showed that low ABI was more common among patients with IS or TIA—51% of participants. The authors justified a larger percentage of patients with low ABI by their higher average age and higher number of atherosclerotic cerebral incidents [16]. The ARIC study found the lowest percentage of low ABI in patients with IS or TIA, which was 9%, but the data may have been underestimated as the measurement was made in one limb only [4]. In the meta-analysis of 17 studies containing 9404 stroke patients aged 64–79 years, a wide range of low ABI from 7.4% to 40.5% was presented. Authors concluded that detection of low ABI increases the risk of myocardial infarct, stroke, and mortality and helps to identify high-risk patients for secondary stroke prevention [28].

Population studies for atherosclerosis in three vascular beds (coronary artery disease, cerebrovascular disease, LEAD) provided very valuable data. In a Global Atherothrombosis Assessment (AGATHA) Study, isolated cerebrovascular disease occurred in 20.4% of the study population. Among this group, low ABI was diagnosed in 26.1% of cases, with a mean value of 0.985 [29]. In our study, the mean value and median ABI (excluding ABI > 1.4) were even lower 0.88 and 0.91; respectively. In a meta-analysis of 10 studies, authors found that ABI < 0.9 was more often associated with stroke (RR 1.83) and concluded that low ABI is an independent risk factor for ischemic stroke [30]. An analysis of data from 6382 adults observed that the risk of stroke increases with a decrease in the value of ABI [31]. Measurement of ABI in patients with ACIE may identify those with second vascular territory involvement. Such individuals could benefit from more aggressive treatment of comorbidities and modification of cardiovascular risk factors.

In this study, there was statistically significant difference between ABI among IS and TIA patients. One study showed a more frequent occurrence of LEAD in stroke patients compared to TIA patients 32% vs. 16.8%, *p* = 0.005, respectively [32]. However, in another study the participants with an ABI <0.8 were more than twice as likely as those with an ABI of 1.0 to <1.5 to have a history of stroke or TIA [33]. The above results compared with our study do not seem surprising, because the risk factors for IS and TIA are the same.

In our study group, the mean/median ABI values between men and women were comparable (0.86/0.88 vs. 0.9/0.92). No sex differences were found in each group in ABI values. In AGATHA Study there were also no sex differences in the group with low ABI (men 37.2% vs. women 34.4%) [29].

In this study, subjects above and below 65 years old in the stroke group had significantly lower ABI than in the controls. Thus, patients may be affected in two vascular territories irrespective of age. In a large population database, there was a strong association between higher age and prevalence of LEAD and carotid artery stenosis (CAS) with odds ratio (OR) 2.14 and 1.80, respectively [15].

### 4.2. LEAD Risk Factors and ABI

Our study showed that the number of active smokers was significantly higher in the stroke group than in the control group; 48 (32%) vs. 8 (16%), *p* < 0.05. Smokers are two to three times more likely to be at risk for stroke. The risk of stroke increases with the number of cigarettes smoked, while the risk may disappear completely after five years of complete cessation of smoking [34]. Smoking is the most important risk factor for LEAD, increasing the risk of LEAD at least 2.5-fold compared to non-smokers [2]. The relative risk of LEAD has also been shown to be 7-times higher among former smokers and 16-times higher among current smokers compared to people who have never smoked cigarettes [35]. The results of large population studies confirm the significant independent relationship between LEAD and smoking [33,36,37]. In our study, in the stroke group, both current and previous smoking influenced ABI values. ABI values were similar between current smokers and past smokers. One study has shown that at least 20 years should pass after smoking cessation to reduce LEAD risk level to compare to non-smokers [38].

The second risk factor with significant impact on lower ABI in the current study was coronary artery disease, which is one of the widely recognized LEAD risk factors. In AGATHA Study, atherosclerosis in the coronary and peripheral beds was 6.7%, and the mean ABI was about 0.7 [29]. Coronary artery disease is a strong and independent risk factor for stroke [39]. In the current study, coronary artery disease was much more common in the stroke group. Data of REGARDS study also confirmed the significant relationship between IS and coronary artery disease [40].

### 4.3. Relationship of ABI and ICA Stenosis

Population-based studies estimate that 15–16% of IS are due to atherosclerosis of large vessels [39,41]. In the present study, we found that the ICA ≥50% stenosis as well as the ICA occlusion occurred more frequently in the stroke group.

When analyzing ACIE patients we found that in the subgroup with ICA stenosis ≥50%, the median ABI was lower than in those without ICA stenosis ≥50%, 0.7 vs. 0.92 *p* = 0.001.

Such relation was described previously by Lee and colleagues who demonstrated lower ABI value in patients with any carotid stenosis (1.04 ± 0.09 vs. 1.13 ± 0.16), *p* = 0.005 [42]. This is also in concordance with studies where authors have found a significant association between CAS and PAD [20,43]. Moreover, the Framingham Study showed that risk of IS or TIA increased with lower ABI value and the HR risk index was 2.2 for ABI <0.9. In patients with ABI 0.9–1.0 and ABI >1.0 the HR was 1.5 and 1.0, respectively [6].

### 4.4. Stroke Subtypes and ABI

The lowest ABI value was in a large-artery atherosclerosis subtype (22.67%), which was not surprising due to coexistence of different vascular bed involvement. In subtype of other undetermined etiology (multifactorial) ABI value was also low. It means that in this subtype coexistence of LEAD was also frequent. Saji et al. compared stroke patients with small-artery disease and those with large-artery atherosclerosis. They found statistically significant difference between those stroke subtypes, but median ABI values were 1.11 (1.05–1.17) in small-artery disease stroke and 1.07 (1.01–1.12) in large-artery atherosclerosis stroke [44]. Ratanakorn et al. showed a significant difference in the prevalence of lower ABI among stroke subtypes, which tended to be more frequent in those with large artery disease (20.4%) and undetermined etiology (20.6%). The same author also found the lower ABI in cardioembolic stroke (29.2%) [25]. In the presented study the authors demonstrated the lower ABI in cardioembolic stroke in 16.67%.

### 4.5. Limitations

In the present study age and sex distribution between stroke group and controls were significantly different. Explanation of these differences is described above. We consider that discrepancy of demographic data had no influence on obtained results. It was confirmed by results of ABI values in relationship between male and female patients as well as younger and older patients.

## 5. Conclusions

Our study demonstrated that the prevalence of low ABI is significantly higher in patients with ACIE (IS or TIA). This suggests that involvement of a second vascular territory presenting as LEAD is common in patients with cerebrovascular events. Coronary artery disease, ICA ≥ 50% stenosis and smoking are some of the major risk factors associated with coexistence of LEAD and ACIE. The study shows the possibility for application of ABI measurement as a first line tool for vascular diseases screening among populations with a high cardiovascular disease burden. Identification of these patients would allow for implementation of earlier primary prevention as well as intensification of secondary prevention after acute cerebral events.

## Figures and Tables

**Table 1:** Demographic data.

	Stroke Group *n* = 150	Control Group *n* = 50	*p*-Value
Age (years)	Mean 67.3 Median 66 (44–92)	Mean 63.36 Median 62 (48–85)	<0.05
Sex (male) (*n*/%)	90/60%	20/40%	<0.05

**Table 2:** Risk factors for an acute cerebral ischemic incident: * IS/TIA—Ischemic stroke/transient ischemic attack.

Risk Factors	Stroke Group No of Patients (%)		Control Group No of Patients (%)		X^2^ Test	*p*-Value
	YES	NO	YES	NO		
Previous IS/TIA *	33 (22)	117 (78)	4 (8)	46 (92)	3.99	<0.05
Hypertension	132 (88)	18 (12)	28 (56)	22 (44)	24	<0.0001
Treated	90 (60)	60 (40)	25 (50)	25 (50)	1.53	NS
Untreated	42 (28)	108 (72)	3 (6)	47 (94)	9.19	<0.01
Atrial fibrillation	29 (19.33)	121 (80.67)	1 (2)	49 (98)	7.53	<0.01
Coronary artery disease	44 (29.33)	106 (70.67)	4 (8)	46 (92)	8.22	<0.001
Hemodynamic significant stenosis ICA ≥ 50%	37 (24.67)	113 (75.33)	0	50 (100)	11.72	<0.001
Diabetes	56 (37.33)	94 (62.67)	6 (12)	44 (88)	11.20	<0.001
Cholesterol total (>200 mg%)	49 (33.56)	101 (66.44)	24 (52.17)	26 (47.83)	3.80	0.51
Triglycerides (>150 mg%)	43 (29.45)	107 (70.55)	16 (34.78)	34 (65.22)	0.20	NS
HDL cholesterol (<40 mg%)	72 (51.43)	78 (48.57)	10 (21.74)	40 (78.26)	12.15	<0.001
LDL cholesterol (>135 mg%)	36 (25.53)	114 (74.47)	19 (40.42)	31 (59.58)	3.69	0.55
Smoking	74 (49.33)	76 (50.67)	21 (42)	29 (58)	0.81	NS
Current	48 (32)	102 (68)	8 (16)	42 (84)	4.74	<0.05
Previous	26 (17.33)	124 (82.67)	13 (26)	37 (74)	1.79	NS

**Table 3:** Distribution of normal and low ankle–brachial index (ABI) values in the stroke and control groups (excluding ABI > 1.4).

ABI	Classification ABI	Stroke Group	Control Group	*p*-Value
		*n* (%)		
≤0.9	Low	69 (46)	8 (16)	0.0001
0.91–1.39	Normal	69 (46)	37 (74)	

**Table 4:** ABI values in the stroke and control groups.

	Stroke Group	Control Group	*p*-Value
ABI	Median (Min–Max)		<0.0001
	0.91 (0.24–1.33)	1.0 (0.66–1.36)	
	Mean (SD)		
	0.88 (SD = 0.22)	1.04 (SD = 0.16)	

**Table 5:** ABI values in relation to sex and age in both groups.

	Stroke Group	Control Group	*p*-Value
	ABI Median (Min–Max)		
Sex			
Male	0.88 (0.24–1.25)	1.08 (0.75–1.36)	<0.001
Female	0.92 (0.53–1.33)	1.0 (0.66–1.25)	<0.01
Age			
≤65 y	0.9 (0.46–1.25)	1.0 (0.75–1.36)	<0.001
>65 y	0.92 (0.24–1.33)	1.07 (0.66–1.25)	<0.05

**Table 6:** ABI values depending on the presence of risk factors in the stroke group.

Risk Factor	YES	NO	*p*-Value
	ABI Median (Min–Max)		
Sex (male)	0.88 (0.24–1.25)	0.92 (0.53–1.33)	NS
Previous IS/TIA *	0.92 (0.25–1.23)	0.88 (0.24–1.33)	NS
Hypertension	0.92 (0.24–1.33)	0.79 (0.48–1.16)	NS
Hypertension treated vs. non treated	0.95 (0.24–1.33) (treated)	0.85 (0.5–1.25) (non-treated)	NS
Atrial fibrillation	0.93 (0.46–1.33)	0.88 (0.24–1.25)	NS
Coronary artery disease	0.85 (0.25–1.23)	0.92 (0.24–1.33)	<0.05
Significant stenosis ICA ≥50%	0.7 (0.25–1.33)	0.92 (0.24–1.25)	<0.001
Diabetes (with ABI >1.4)	1.0 (0.24–2.5)	0.92 (0.25–1.6)	NS
Cholesterol total (>200mg%)	0.92 (0.4–1.25)	0.9 (0.24–1.23)	NS
Triglycerides (>150mg%)	0.92 (0.24–1.33)	0.9 (0.25–1.23)	NS
HDL cholesterol (<40mg%)	0.91 (0.24–1.25)	0.89 (0.25–1.25)	NS
LDL cholesterol (>135mg%)	0.92 (0.46–1.33)	0.9 (0.24–1.25)	NS
Smoking (current and previous)	0.83 (0.24–1.25)	0.98 (0.53–1.33)	<0.001
Smoking Current vs. previous	0.82/0.83 (0.24–1.25) Current	0.79/0.76 (0.25–1.23) Previous	NS

* IS/TIA—Ischemic stroke/transient ischemic attack.

**Table 7:** Distribution of ABI values among stroke subtypes.

Stroke Subtype (%)	ABI Value Median (min–max)
Large-artery atherosclerosis (stenosis >50%) (22.67)	0.71 (0.25–1.33)
Cardioembolism (16.67)	0.93 (0.48–1.21)
Small-vessel occlusion (20.00)	1.06 (0.53–2.5)
Stroke of undetermined etiology (40.67)	0.9 (0.24–1.45)

Kruskal–Wallis test, *p* < 0.0001.
